# The impact of temporal variability of biochemical markers PAPP-A and free β-hCG on the specificity of the first-trimester Down syndrome screening: a Croatian retrospective study

**DOI:** 10.1186/1756-0500-3-194

**Published:** 2010-07-14

**Authors:** Dubravka Tišlarić-Medenjak, Ivana Zec, Ana-Maria Šimundić, Senka Sabolović-Rudman, Milan Kos, Željka Bukovec Megla

**Affiliations:** 1 Laboratory of Endocrinology, Clinics of Oncology and Nuclear Medicine, University Hospital "Sestre milosrdnice", Zagreb, Croatia; 2 University Department of Chemistry, University Hospital "Sestre milosrdnice", Zagreb, Croatia; 3 Clinics of Gynecology and Obstetrics, University Hospital "Sestre milosrdnice", Zagreb, Croatia; 4 Out-patient Clinics of Gynecology "VILI", Zagreb, Croatia

## Abstract

**Background:**

The variability of maternal serum biochemical markers for Down syndrome, free β-hCG and PAPP-A can have a different impact on false-positive rates between the 10+0 and 13+6 week of gestation. The study population comprised 2883 unaffected, singleton, spontaneously conceived pregnancies in Croatian women, who delivered apparently healthy child at term. Women were separated in 4 groups, dependently on the gestational week when the analyses of biochemical markers were performed. The concentrations of free β-hCG and PAPP-A in maternal serum were determined by solid-phase, enzyme-labeled chemiluminiscent immunometric assay (Siemens Immulite). Concentrations were converted to MoMs, according to centre-specific weighted regression median curves for both markers in unaffected pregnancies. The individual risks for trisomies 21, 18 and 13 were computed by Prisca 4.0 software.

**Findings:**

There were no significant differences between the sub-groups, regarding maternal age, maternal weight and the proportion of smokers. The difference in log_10 _MoM free β-hCG values, between the 11^th ^and 12^th ^gestational week, was significant (p = 0.002). The difference in log_10 _MoM PAPP-A values between the 11^th ^and 12^th^, and between 12^th ^and 13^th ^week of gestation was significant (p = 0.006 and p = 0.003, respectively). False-positive rates of biochemical risk for trisomies were 16.1% before the 11^th ^week, 12.8% in week 12^th^, 11.9% in week 13^th ^and 9.9% after week 13^th^. The differences were not statistically significant.

**Conclusions:**

Biochemical markers (log_10 _MoMs) showed gestation related variations in the first-trimester unaffected pregnancies, although the variations could not be attributed either to the inaccuracy of analytical procedures or to the inappropriately settled curves of median values for the first-trimester biochemical markers.

## Findings

The first-trimester screening for trisomies 21, 18 and 13, combining maternal age, fetal nuchal translucency thickness (NT), maternal serum free β-human chorionic gonadotropin (free β-hCG) and pregnancy associated plasma protein-A (PAPP-A) has revealed a highly potential possibility of an early detection of fetal aneuploidies [1-5].

Various co-variables influence the specificity and sensitivity of the first-trimester biochemical markers have been studied [6]. Corrections of the calculated gestational MoM values (Multiples of the Median) have been proposed for maternal weight, cigarette consumption, ethnicity and twin-pregnancy [7-10]. Fetal gender, maternal insulin-dependent diabetes mellitus, parity/gravidity and procedures in assisted reproduction show certain, but insignificant influence on the screening performance [11-15].

Several studies have focused on the optimal timing for analysis of biochemical markers, predominantly concerning their sensitivity for fetal trisomies [16,17]. The optimal sensitivity of PAPP-A is between 9-12 weeks' gestation, being significantly reduced towards the 13^th ^week [18]. Clinical sensitivity of free β-hCG becomes evident from the 10^th ^week, ongoing towards the second trimester of pregnancy [19].

To our knowledge, there has been a lack of publications, which presume the precision of the applied analytical methods to the physiological variations of maternal serum markers across the first trimester. This retrospective study in Croatian women with unaffected pregnancies has the aim to: (a) assess the specificity of free β-hCG and PAPP-A across the first-trimester in terms of the false-positive rate (FPR) for trisomy 21 in particular weeks and (b), to examine the analytical accuracy of the methods applied for the measurement of biochemical markers.

## Methods

In Croatia, combined screening for fetal trisomies has been performed from February 2006. Our Laboratory has been the only centre, which has conducted the analyses for biochemical markers according to the recommendations of the Fetal Medicine Foundation (www.fetalmedicine.com). Also, we have been included in external proficiency testing program (UK NEQAS First Trimester Down Syndrome Screening) since August 2006.

Out of total 3118 pregnant women who underwent the combined screening for chromosomal abnormalities till May 2008, a cohort of 2883 subjects was retrospectively recruited from the entire population. The selection criteria included non-diabetic, Caucasian women, with singleton, spontaneously conceived pregnancy, who delivered apparently healthy child at term. Pregnancies with abnormal fetal karyotype and those spontaneously aborted were excluded, on the basis of the data supplemented from health-care providers and delivery records in maternity units, respectively.

All pregnant women had an ultrasound examination between weeks 10+0 and 13+6, which included the crown-rump (CRL) measurement for pregnancy dating and the correspondent NT value, respectively. The obstetrician filled the form with all the clinical data relevant for the pregnancy and the mother. The Hospital Ethics Committee approved the accessibility of the first-trimester screening to all pregnant women (E.C. No.19-1/2006). Each woman signed informed consent for the combined screening.

The concentrations of maternal serum free β-hCG and PAPP-A were determined by solid-phase, enzyme-labelled chemiluminiscent immunometric assay (Siemens Medical Solution Diagnostics). Assays were performed on automated platform Immulite 1000. Median levels of free β-hCG and PAPP-A in unaffected pregnancies were calculated from weighted regression of observed medians for each gestational day. For that purpose, pregnancies achieved by the methods of assisted conception, twins, diabetic pregnant women and pregnancies with confirmed fetal aneuploidies were excluded from the database. The data fitted the exponential equation well; observed medians were close to those predicted from the model. Regressed medians were used to generate multiples of the median (MoM) for each case. MoM values of markers were corrected for maternal weight and smoking cigarettes and individual risks for trisomies 21, 18 and 13 for each of the pregnant women were calculated using the Prisca 4.0 software (Typolog, Germany).

To assess the accuracy of the analytical procedures, specimens of Siemens control modules LPCCM and LFBCM were run in a day-routine (three-level control containing free β-hCG in the lyophilized serum preparation and two-level control for PAPP-A in non-human serum matrix, respectively). To examine the within-run precision, 15 replicates of each control were analyzed sequentially. To evaluate the between-day precision, an aliquot of the same lot of control specimen was run along with daily routine measurements. In addition, an aliquot of pooled pregnant women sera was run with daily batch of unknown samples.

The impact of variability of biochemical marker MoM values on the risk for trisomy 21 was assessed using the appropriate 15 paired concentrations of free β-hCG and PAPP-A in controls and in human pooled sera as well, in combination with a fixed clinical pattern (CRL, NT, maternal age and maternal weight) and the relevant risks were computed.

Statistical analyses were performed using MedCalc^® ^software and descriptive statistics using Excel 7.0, respectively. The normality of distributions of log_10 _MoMs of biochemical markers was ascertained using Kolgomorov-Smirnov test. Proportions of smokers and FPR in particular weeks were determined by *χ*^2 ^test. The differences in maternal age and maternal weight, between particular weeks, were assessed by one-way ANOVA. The level of p < 0.05 was considered statistically significant.

## Results

The data concerning within-run and between-day analytical precision for the biochemical markers are presented in table 1. Table 2 shows the within- and between-day variations of biochemical and combined risk, in the three levels of risk values. The within-day coefficient of variation (CV) varied between 9.45-11.72% for biochemistry only and between 10.45-12.98% for combined risk, respectively. The between-day variations of the biochemical risk were between 10.39-12.47%, and of the combined risk between 11.02-12.77%, depending on the particular risk level.

**Table 1:** Within-run and between-day precision of measurement of free β-hCG and PAPP-A, using Siemens Control Modules and human pooled sera on automated platform Immulite 1000

		Free β-hCG (ng/ml)			PAPP-A (mIU/ml)		
**Sample**	**Control Module LFBCM**			**Human serum**	**Control Module LPCCM**		**Human serum**
				
	**Control 1**	**Control 2**	**Control 3**		**Control 1**	**Control 2**	

**Target**	5.1	23.8	74.2		0.40	1.96	
2SD Range	(4.2-6.4)	(19.8-27.8)	(63.6-90.8)		(0.32-0.48)	(1.70-2.22)	

**Within-run**							
Mean	4.84	24.1	72.8	66.6	0.41	1.94	2.58
SD	0.15	1.12	3.50	2.40	0.01	0.06	0.17
CV* (%)	3.09	4.65	4.81	3.60	2.44	3.09	6.59

**Between-run**							
Mean	4.75	22.66	71.66	61.7	0.38	1.94	2.39
SD	0.31	1.70	5.25	4.13	0.02	0.11	0.21
CV* (%)	6.52	7.50	7.32	6.69	5.26	5.67	8.79

CV* - coefficient of variation

**Table 2:** Impact of within-day and between day variations on assessment of biochemical and combined risk for Down syndrome

		Free β-hCG MoM	PAPP-A MoM	Biochemical risk (1 in)	Combined risk (1 in)
**Within-day variations of calculated risk**	High risk *				
	Mean ± SD	1.87 ± 0.08	0,29 ± 0,01	52.6 ± 6.2	154.4 ± 16.1
	CV† (%)	4.27	3.45	11.72	10.45
	
	Medium risk **				
	Mean ± SD	0.59 ± 0,03	0,29 ± 0,01	732.6 ± 76.0	699.9 ± 77.2
	CV† (%)	5.08	3.45	10.37	11.03
	
	Low risk ***				
	Mean ± SD	1.87 ± 0.08	1.26 ± 0.05	3060.8 ± 323.5	2527.7 ± 328.3
	CV† (%)	4.27	3.97	10.57	12.98
	
	**Human serum**				
	Mean ± SD	1.47 ± 0.07	1.79 ± 0.06	8668.2 ± 819.4	7313.9 ± 900.9
	CV† (%)	4.76	3.35	9.45	12.32

**Between-day variations of calculated risk**	High risk *				
	Mean ± SD	1.70 ± 0.09	0.27 ± 0.01	45.39 ± 5.5	145.6 ± 18.2
	CV† (%)	5.26	3.43	12.12	12.50
	
	**Medium ris**k **				
	Mean ± SD	0.59 ± 0.03	0.27 ± 0.01	661.4 ± 68.7	646.1 ± 68.58
	CV† (%)	4.3	3.43	10.39	11.54
	
	Low risk ***				
	Mean ± SD	1.70 ± 0.09	1.26 ± 0.05	2442.6 ± 304.6	1993.3 ± 219.7
	CV† (%)	5.26	4.28	12.47	11.02
	
	**Human serum**				
	Mean ± SD	1.36 ± 0.08	1.76 ± 0.09	7446.5 ± 796.8	6146.4 ± 785.4
	CV† (%)	6.17	5.20	10.70	12.77

*Biochemistry: Control 3 (free β-hCG) + Control 1 (PAPP-A); Patient: 25 years, non-smoker, 67 kg, CRL = 45 mm, NT = 1.5 mm

**Biochemistry: Control 2 (free β-hCG) + Control 1 (PAPP-A); Patient: 25 years, non-smoker, 67 kg, CRL = 45 mm, NT = 1.8 mm

***Biochemistry: Control 3 (free β-hCG) + Control 2 (PAPP-A); Patient: 25 years, non-smoker, 67 kg, CRL = 45 mm, NT = 1.8 mm

CV† - coefficient of variation

To investigate a possible gestation-dependent biological variability of biochemical markers, the pregnant women were classified in 4 groups, according to gestational age at blood sampling. There were no significant differences between the sub-groups, regarding maternal age, maternal weight and the proportion of smokers (table 3).

**Table 3:** Distribution of pregnancies through gestation according to demographic characteristics

Group	Week of gestation	Number	Maternal age (years)* Mean ± SD	Maternal weight (kg) ** Mean ± SD	Smokers *** N (%)
**1**	10 to 10+6/7	96	31.7 ± 4.5	66.9 ± 12.8	10 (10.4%)

**2**	11 to 11+6/7	853	31.5 ± 4.6	66.4 ± 11.2	91 (10.7%)

**3**	12 to 12+6/7	1326	31.4 ± 4.4	65.4 ± 10.6	153 (11.5%)

**4**	13 to 13+6/7	608	31.4 ± 4.4	65.6 ± 10.4	61 (10.0%)

	**Total**	**2883**	**31.5 ± 4.5**	**66.1 ± 11.3**	**315 (10.9%)**

* p = 0.834 (one-way analysis of variance)

** p = 0.124 (one-way analysis of variance)

*** p = 0.779 (*χ*^2 ^=1.093)

The distribution of maternal serum free β-hCG and PAPP-A MoM values, according to gestational age, are presented in table 4. Median MoMs of both markers were close to 1.0 in all of four weeks of gestation. For the both analytes and in all of the weeks, log_10 _MoM values fitted the Gaussian distribution, with overall standard deviation for free β-hCG of 0.2600, and 0.2307 for PAPP-A, respectively.

**Table 4:** Distribution of maternal serum free β-hCG and PAPP-A MoM values, according to gestational age

		Free β-hCG MoM					PAPP-A MoM		
		
Group	Week of gestation	Median	Mean **log**_**10**_	SD **log**_**10**_	CV* (%)	Median	Mean log_10_	SD **log**_**10**_	CV* (%)
**1**	10 to 10+6/7	1.04	-0.0655	0.2494	0.04	0.99	0.0043	0.2398	0.55

**2**	11 to 11+6/7	0.98	0.0294	0.2651	0.09	0.98	-0.0177	0.2204	0.12

**3**	12 to 12+6/7	0.99	0.0334	0.2619	0.08	0.99	0.0170	0.2335	0.14

**4**	13 to 13+6/7	1.03	0.0294	0.2637	0.09	1.02	0.0453	0.2290	0.05

	**Total**	**1.00**	**0.0067**	**0.2600**	-	**0.98**	**0.0122**	**0.2307**	-

Gaussian distribution was confirmed by Kolgomorov-Smirnov test for normality

MoM - Multiples of the Median

CV* - coefficient of variation

Table 5 presents the results of assessment of variability for log10 MoM values of free β-hCG and PAPP-A between particular weeks. Using ANOVA, we showed statistically significant difference in log_10 _MoM values of free β-hCG between the 11^th ^and 12^th ^gestational week (p = 0.002). In addition, significant differences were scertained for log_10 _MoM PAPP-A between the 11^th ^and 12^th^, as well as between 12^th ^and 13^th ^week of gestation (p = 0.006 and p = 0.003, respectively). Post-hoc Student-Newman-Keuls test was used to assess the significance of the difference between subgroups.

**Table 5:** Variability of log_10 _MoM values of free β-hCG and PAPP-A between particular weeks

		Free β-hCG			PAPP-A
		
Between-weeks' difference	Sample size	F-ratio	Significance level	F-ratio	Significance level
Group 1 vs. Group 2 (10+0 to 11+6)	949	9.221	p = 0.002*	0.191	p = 0.294

Group 2 vs. Group 3 (11+0 to 12+6)	2179	0.0372	p = 0.847	7.445	p = 0.006*

Group 3 vs. Group 4 (12+0 to 13+6)	1934	0.947	p = 0.331	8.942	p = 0.003*

**All**	2883	3.516	p = 0.015*	8.696	p < 0.001*

* Statistically significant (one-way analysis of variance)

In table 6, we compared the FPR of biochemical risk for trisomies in particular weeks of first-trimester gestation, considering the cut-off values 1:300 for trisomy 21 and 1:100 for trisomies 18 and 13, respectively. The proportion of pregnant women, classified at increased biochemical risk, was 16.1% in the group before the 11^th ^week, 12.8% between weeks 11 and 11+6, 11.9% between weeks 12 and 12+6, and 9.9% between weeks 13 and 13+6, respectively. The differences in proportions were not statistically significant, neither for the risks concerning trisomy 21 nor for trisomies 18/13.

**Table 6:** Comparison of the false positive rates (FPR) in calculated biochemical risk in particular weeks

Group	Week of gestation	Risk for trisomy 21 (Cut-off ≤1:300) N/Total (%)	Risk for trisomy 18 (Cut-off ≤1:100) N/Total (%)	Between-weeks' difference *
**1**	10 to 10+6/7	15/96 (16.1%)	2/96 (2.2%)	/

**2**	11 to 11+6/7	109/853 (12.8%)	17/853 (2.0%)	Group 1 vs. Group 2 *χ*^2^=0.402, p = 0.52

**3**	12 to 12+6/7	158/1326 (11.9%)	15/1326 (1.1%)	Group 2 vs. Group 3 *χ*^2^=0.313, p = 0.58

**4**	13 to 13+6/7	60/608 (9.9%)	10/608 (1.6%)	Group 3 vs. Group 4 *χ*^2^=0.813, p = 0.37

* The differences are not significant (Data are referring to calculated Down syndrome biochemical risk)

## Discussion

Introducing the first-trimester screening for fetal trisomies in Croatian pregnant women has been the opportunity for the improvement of prenatal care in our country [20,21]. Previous studies have reported considerable variations in analytical procedures and precision of analyte measurement from different laboratories providing biochemical analyses [22]. That refers not only to the accuracy of measured concentrations of free β-hCG and PAPP-A, but also to converting them to the appropriate MoM values, and as the consequence, estimating the computed risks for fetal aneuploidies. In our country we have quite uniform population of women, concerning their demographic, social and ethnic background [20]. In that way, we have the opportunity to create the stable population median values that produce correct MoMs and derive the results of the biochemistry from a single Laboratory. The MoM values for both analytes were close to 1.00 in each of the weeks, suggesting that the applied medians have been appropriate. We also have proved that the distribution of log_10 _MoM values of both markers fits a Gaussian curve, with SDs comparable with the results of much greater series in the literature [1-5]. Our results undoubtedly demonstrated the precision of the measurement of biochemical markers. Coefficients of intra- and inter-assay variations for both markers were exactly as proposed by the manufacturer. Concerning the fact that all analytical procedures were performed on automatic Immulite 1000 system, the data suggest more accurate and precise measurement performance than Spencer demonstrated when he had evaluated Immulite 2000 versus Kryptor platform [23]. He established a significant positive bias for PAPP-A (20.65%) against the ALTM of UK NEQAS for first-trimester screening. As of our experience, we disagree with this statement, since our Laboratory participates with the Immulite technology in the same program. The proceedings of NEQAS have demonstrated that our BIAS in PAPP-A concentrations, against ALTM, has been -1.8% (VAR 4.8%), while BIAS for MoM values have been -12.6% (VAR 9.4%). On the other side, BIAS for free β-hCG has been -1.7% (VAR -4.1%) and for MoM values +1.4% (VAR +4.1%), respectively. Most probably, some considerable analytical improvements may have occurred for free β-hCG and PAPP-A immunoassays on Immulite platforms since the year 2005, when Spencer published his study. Nonetheless, we can confirm that we have succeeded in keeping the high technical standards for biochemical marker analyses [24]. In line with Spencer's recommendations, the assessment of calculated risk variability is an obligatory and useful tool in quality control [25]. We have shown that within-day and between-run CVs of calculated risks were, in rough, between 10-13%. This is to some extent higher than 8%, which is the optimal CV for target risk, but we need to continue with this monitoring for a longer period to have the more representative experience. On the other hand, proper risk classification of pregnant women can be influenced not only by biochemical markers, but even more, by the imprecise measurement of ultrasonic markers, which is outside the scope of this study.

From the point of clinical interest, the most important is the discriminatory power of both biochemical markers across the first-trimester. The impact of variability of free β-hCG and PAPP-A on their clinical sensitivity was clearly demonstrated in studies of Spencer and his co-workers [26,27].

To examine biological variations in normal pregnancies, we studied the variability of marker concentrations and their MoM values across the first trimester. The uniformity of sub-groups in particular weeks was proved by means of ANOVA and *χ*^2 ^test. The compliance of log_10 _MoM values with normal distributions, and insignificant changes of SDs of both markers in particular weeks did not show any significant oscillations of the values of free β-hCG and PAPP-A. When we compared log-transformed MoM values between weeks by ANOVA, statistically significant differences were found for free β-hCG MoM values between the first two weeks. On the other side, significant differences for PAPP-A MoM values were established between sub-groups in later weeks of the first trimester.

Spencer and co-workers determined the optimal time for PAPP-A measurement between 8-10 weeks' gestation, while its detection efficiency gradually decreased from 10 to 13 weeks' gestation. On the other side, the discriminatory power of free β-hCG was the best after the 12^th ^week [26]. Our study hasn't been designed to prove the best evidence, of neither of biochemical markers, in identifying the affected pregnancies. Small number of Down syndrome cases in study population would not allow the meaningful statistical analysis. The aim of this investigation was to discern a period in the first-trimester when the biochemical markers are most specific and if FPR, due to biochemistry, differ between the particular weeks. In our pregnant women, the proportion of those at "high biochemical risk" decreased from the week 10+0 to week 13+6, although the differences were not statistically significant. This study was unfortunately underpowered to detect such difference as significant. Over 1,000 pregnant women per week are needed to detect the observed difference as significant, with the power of 0.8.

We think that it is important to get better insight in possible physiological variations of the first-trimester biochemical markers in particular weeks and to study the degree of the oscillations of mean marker values between the constant ranges of respective standard deviations. Although the number of our pregnant women was not large enough to form representative sub-groups, we have clearly shown that the observed variations are not caused by inaccuracy of analytical procedures or by a bias produced by inappropriate median values of biochemical markers across the gestational range. Considering the prevailing standards for analytical laboratories, this is of value for correct interpretation of the results of biochemical markers, particularly concerning their specificity.

## Conclusions

We have shown the gestation related variations of log_10 _MoM values for biochemical markers, across the first-trimester weeks in unaffected pregnancies. According to our results, it seems unlikely that the observed variations could be attributed either to inaccuracy of analytical procedures or to inappropriately settled curves of median values for the first-trimester biochemical markers. Improvement of the screening strategy implies, on one side, following the guidelines of the Fetal Medicine Foundation and, on the other, a better setting of the timing for the biochemical parameters, for it can contribute to a higher specificity of the screening.

## Competing interests

The authors declare that they have no competing interests. Patients' data relevant for this study were retrospectively extracted from the routine praxis and no other financial funds were employed.

## Authors' contributions

DTM has been the main co-ordinator of screening and conceived the study. IZ carried out analytical procedures and performed all statistical analyses. AMS participated in design of study and revised the manuscript. SSR and MK performed most of ultrasound examinations. ZBM helped in acquisition of data. All authors read and approved the final version of manuscript.
